# A Case of Epilepsy Suspended by a Burn on the Sole of the Foot

**Published:** 1829

**Authors:** H. E. Green

**Affiliations:** Greenupsburgh, Kentucky


Art. IV.—*A case of Epilepsy suspended by a Burn on the sole of the Foot.* Communicated by Dr. H. E. Green, of Greenupsburgh, Kentucky.

On the 5th of October, 1827, I was applied to for advice in a case of violent Epileptic fits. The subject was a colored man and a slave, about 48 years old, and of steady habits. It was about three months since the first attack; but he had complained for 12 or 18 months previously, of tension, uneasiness and pain about the umbilical region, espcially after eating a full meal. He had, all this time, done the work of a common laborer. The convulsions came on without any previous notice, at each full and change of the moon, and he would have from four to ten at each attack. When I first saw him, he was moderately corpulent, and looked healthy; he said, however, that he was in a very costive habit, and frequently went two or three days without an evacuation.

I bled him and ordered it to be repeated every 10 or 12 days for two months; inserted a large issue in the nape of his neck; directed the daily use of pills composed of aloes, ipecacuanha, and the blue mercurial mass, and prescribed a simple diet. This course was continued for three months without any sensible variation in the disease. He then took three grains of the nitrate of silver, daily, for two months, with no better success. He now fell into the hands of a *patent steam doctor*, by whose engine he was nearly destroyed.

At length all hope of relief was despaired of, when in the month of January, 1829, in a severe fit, alone, he fell into the fire and *burnt severely the whole of the bottom of the left foot.* It

*did not get well for about four months, during the whole of which time he had no fits, but exhibited every indication of returning health and vigour. As soon, however, as it was healed, the fits returned.*

Profiting by the hint afforded by this accident, I have put an issue into each ankle, the effect of which remains to be ascertained.

*July,* 1829.

